# Structural and mechanical properties of the red blood cell’s cytoplasmic membrane seen through the lens of biophysics

**DOI:** 10.3389/fphys.2022.953257

**Published:** 2022-09-12

**Authors:** Sebastian Himbert, Maikel C. Rheinstädter

**Affiliations:** ^1^ Department of Physics and Astronomy, McMaster University, Hamilton, ON, Canada; ^2^ Origins Institute, McMaster University, Hamilton, ON, Canada

**Keywords:** red blood cells, RBCs, RBC membrane, RBC biophysics, RBC mechanical properties, RBC membrane structure, RBC cytoplasmic membrane

## Abstract

Red blood cells (RBCs) are the most abundant cell type in the human body and critical suppliers of oxygen. The cells are characterized by a simple structure with no internal organelles. Their two-layered outer shell is composed of a cytoplasmic membrane (RBC_*cm*_) tethered to a spectrin cytoskeleton allowing the cell to be both flexible yet resistant against shear stress. These mechanical properties are intrinsically linked to the molecular composition and organization of their shell. The cytoplasmic membrane is expected to dominate the elastic behavior on small, nanometer length scales, which are most relevant for cellular processes that take place between the fibrils of the cytoskeleton. Several pathologies have been linked to structural and compositional changes within the RBC_*cm*_ and the cell’s mechanical properties. We review current findings in terms of RBC lipidomics, lipid organization and elastic properties with a focus on biophysical techniques, such as X-ray and neutron scattering, and Molecular Dynamics simulations, and their biological relevance. In our current understanding, the RBC_*cm*_’s structure is patchy, with nanometer sized liquid ordered and disordered lipid, and peptide domains. At the same time, it is surprisingly soft, with bending rigidities *κ* of 2–4 k_B_T. This is in strong contrast to the current belief that a high concentration of cholesterol results in stiff membranes. This extreme softness is likely the result of an interaction between polyunsaturated lipids and cholesterol, which may also occur in other biological membranes. There is strong evidence in the literature that there is no length scale dependence of *κ* of whole RBCs.

## Introduction

**FIGURE 1:**
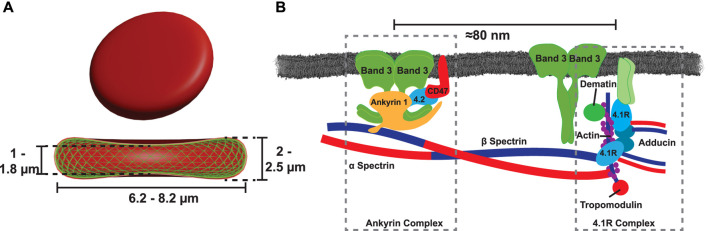
**(A)** Healthy RBCs have a discocyte shape, which assembles the minimal elastic energy of the RBC’s shell. This two layered shell is comprised of a cytoplasmic membrane tethered to a spectrin-based cytoskeleton. **(B)** The cytoplasmic membrane is tethered to the cytoskeleton via two protein complexes, the ankyrin-based complex, and the 4.1R-based complex (Gallagher, 20052005).

Red blood cells (RBCs) are critical suppliers of oxygen to tissues and transport waste carbon dioxide. This unique cell-type thus plays a crucial role in the mammalian metabolism. Healthy RBCs have a biconcave shape, i.e., they appear as round disks with a diameter of ≈ 7 *μ*m and a central dimple under the microscope (as sketched in Figure 1A). Several pathologies, such as sickle cell anemia (Herrick, 1910; Rees et al., 2010), hereditary spherocytosis (Perrotta et al., 2008) or hereditary elliptocytosis (Dresbach, 1904; Palek, 1985), but also infectious diseases, such as malaria (Mohandas and An, 2012), are known to alter the shape of the cells. Unlike most other human cells, RBCs lack complexer internal structures, such as a nucleus or mitochondria, in favor of a larger volume for the oxygen binding protein, hemoglobin.

A particularly long debated question concerns the shape of RBCs. In equilibrium, the biconcave shape must correspond to the total minimum elastic energy of the RBC’s outer shell for a given volume and surface area (Viallat and Abkarian, 2014). This outer shell is comprised of two layers as illustrated in Figure 1B: a cytoplasmic membrane (RBC_*cm*_) and a spectrin-based cytoskeleton (Liu et al., 1987). The RBC’s cytoskeleton is a two-dimensional structure. It is formed by triangularly arranged spectrin filaments parallel to the RBC_*cm*_. The distance between tethers is ≈ 80 nm (Liu et al., 1987). The cytoskeleton is anchored to the RBC_*cm*_ through tethering sites that are formed by two macromolecular complexes of membrane proteins, the ankyrin-based complex, and the 4.1R-based complex (Mohandas and Gallagher, 2008). A schematic illustration of the molecular tethering sites is shown in Figure 1B. Both complexes are highly mobile within the lipid bilayer and allow the RBC_*cm*_ to slide against the cytoskeleton (Discher et al., 1994; Viallat and Abkarian, 2014). Although the cell’s discocyte shape represents the minimal state of the shell’s elastic energy, the cytoskeleton is not considered to be stress free (Viallat and Abkarian, 2014): the spectrin network is believed to be slightly stretched in the resting biconcave shape and to exert a compression force on the lipid bilayer (Sens and Gov, 2007; Viallat and Abkarian, 2014). The cytoplasmic membrane is typically described using the fluid mosaic model (Singer and Nicolson, 1972), which pictures this structure as a two-dimensional fluid-like lipid bilayer with embedded proteins. More than 50 of these membrane proteins have been characterized for the RBC_*cm*_ (Mohandas and Gallagher, 2008).

## The RBC lipidome and pathological lipidomic changes

**FIGURE 2:**
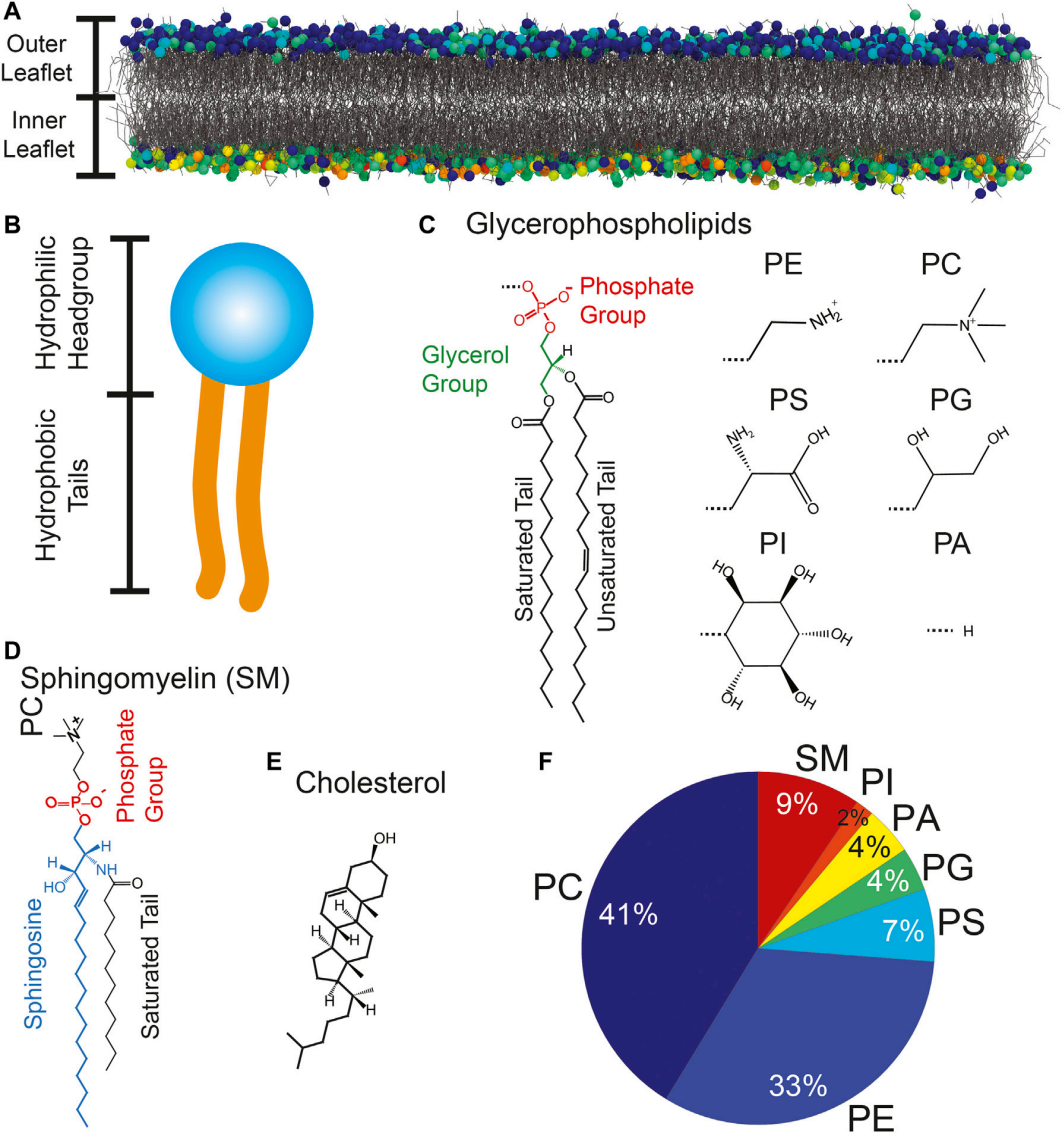
**(A)** The RBC_*cm*_is formed by two leaflets of lipids molecules. **(B)** Glycerophospholipids and sphingomyelin consist of a hydrophilic head group and two hydrophobic tails. **(C–E)** Chemical structures of glycerophospholipids, sphingomyelin and cholesterol. Common head groups are: Phosphatidylethanolamine (PE), Phosphatidylcholine (PC), Phosphatidylserine (PS), Phosphatidylglycerol (PG), Phosphatidylinositol (PI), and Phosphatidic Acid (PA). **(F)** Lipid distribution of the RBC cytoplasmic membrane not including cholesterol.

The lipid bilayer is a ≈ 5 nm (Himbert et al., 2017) thick membrane formed by two lipid layers (leaflets). A three-dimensional render of a Molecular Dynamics (MD) simulation of a RBC_*cm*_ is shown in Figure 2A. There is a variety of different lipids found within the RBC_*cm*_. Lipid molecules are amphiphilic and appear in a wide range of molecular structures giving rise to a manifold of influences onto the membrane’s biophysical properties. Notable examples include glycerophospholipids (PL), sphingomyelin (SM) and cholesterol. The chemical composition of these molecules is well known to the scientific community for more than a century.

The molecular structure of PL and SM is characterized by a hydrophilic head group and flexible hydrophobic tails (Figure 2B). PLs are built around a glycerol moiety as depicted in Figure 2C. The majority of PLs have two fatty acid tails. Lysophospholipid (LPL) are produced by removing one of the tails through hydrolysis. The structure of SM is visualized in Figure 2D. This molecule is built around sphingosine with an attached fatty acid chain and a phosphatidylcholine (PC) head group. The fatty acid tails can vary in length and degree of saturation. Unlike PL and SM, cholesterol is dominated by a rigid structure formed by hydrocarbon rings (Figure 2E). It can account for up to 50  mol% (Dodge and Phillips, 1967) of the membrane’s lipid content, and typically aligns itself upright along the bilayer normal. The lipidome of the RBC_*cm*_ was first determined by Dodge & Phillips in 1967 using gas-liquid chromatography (Dodge and Phillips, 1967). Modern mass spectroscopy experiments allow for high throughput analysis (D’Alessandro et al., 2021; Reisz et al., 2019; Stefanoni et al., 2020; Thomas et al., 2020a). Figure 2F shows the contribution of most lipid species not including cholesterol. PC ( ≈ 40%) and phosphatidylethanolamine (PE) ( ≈ 30%) PLs are the most abundant molecules in the membrane followed, by SM ( ≈ 9%) (Dodge and Phillips, 1967; Stefanoni et al., 2020; Himbert et al., 2021). Phosphatidylserine (PS), phosphatidylglycerol (PG), phosphatidylinositol (PI) and phosphatidic acid (PA) lipids account for ≈ 20% of the membrane. Particularly important to the membrane’s biophysical structure and especially its mechanical properties is also the level of saturation of the fatty acids within the membrane. Most fatty acid tails contain between 16 and 18 methylene moieties but tails with as little as 8 and as much as 36 CH_2_ groups have been detected (Dodge and Phillips, 1967; Stefanoni et al., 2020; Himbert et al., 2021). About 40% of the tails are unsaturated. 21% have one or two double bonds, and 29% have more than three double bonds.

Pathological changes in the RBC_*cm*_’s lipidomic have been widely described in the literature. Excellent reviews of RBC_*cm*_ related diseases can be found in (Gallagher, 2005) and (Narla and Mohandas, 2017). Both, oxidative stress and mechanical stress have been recognized to impair the RBC_*cm*_. They lead to lipid oxidation and may trigger phagocytosis and the consequent clearance of damaged RBC. This process is sometimes referred to as eryptosis (Föller et al., 2008) and is characterized by increased intracellular Ca^2+^, microparticle release and cell shrinkage (Nemkov et al., 2021). Microparticle release is the emission of small vesicles into the blood. Among others, increased microparticle concentration is a key marker for prolonged eryptosis and has been recognized to affect the RBC_*cm*_’s composition (Nemkov et al., 2021). Sickle cell anemia is an inherited genetic disease that causes the formation of hemoglobin fibrils resulting in a loss in the cells’ ability to transport oxygen. Abnormal membrane lipid composition in sickle cell RBCs has been observed (Lubin et al., 1981; Franck et al., 1985; Connor et al., 1997), and was correlated with increased intracellular calcium (Eaton et al., 1978), increased binding of hemoglobin (Asakura et al., 1977), enhanced flip-flop of PC and the exposure of PS on the outer leaflet (Franck et al., 1985), and enhanced susceptibility of sickled erythrocytes to lipid peroxidation (Chiu and Lubin, 1979).

Aside from genetic diseases, infectious diseases have also been reported to alter the composition of the RBC_*cm*_. A particularly important, RBC targeting parasite, is malaria (Mohandas and An, 2012). Fundamental differences between erythrocytes infected with the different parasite stages were revealed. In mature gametocytes many lipids that decrease in the trophozoite and early gametocyte infected RBCs are regained (Tran et al., 2016). In particular, regulators of membrane fluidity, cholesterol and sphingomyelin, increased significantly during gametocyte maturation (Tran et al., 2016). Neutral lipids increased from 3% of total lipids in uninfected to 27% in stage V gametocyte infected RBCs. PL content decreased during gametocyte development (Tran et al., 2016).

Very recently, effects of the SARS-CoV-2 virus onto the concentrations of circulating fatty acids in the blood plasma were reported (Thomas et al., 2020b). Importantly, elevated levels of polyunsaturated fatty acids (PUFAs) were related to an increased activity of phospholipases A2 which is required for viral replication (Müller et al., 2018; Thomas et al., 2020b). An inhibition of phospholipases A2 activity was suggested to reduce disease severity (Thomas et al., 2020b).

Alterations to the RBC_*cm*_’s composition can also be non-pathological. Exercising increases both, shear stress and oxidative stress, but changes of the RBC_*cm*_ strongly depend on the exercise intensity. Short maximal exercise tests do not show signs of microvesicle release (Nader et al., 2018; Tomschi et al., 2018). Ten kilometer running trials also did not elevate eryptosis markers in endurance-trained athletes (Tripette et al., 2011). However, high-intensity 30 min cycling tests showed an increased generation of microparticles even in well-trained subjects (Nader et al., 2020). Consequently, a decrease in several lipids such as LPC(18:1), PC(36:5), SM(42:2), LPC(18:3), LPC(20:5) were observed (Nemkov et al., 2021). Quite interestingly, these were predominantly lipids with polyunsaturated fatty tails.

## Lipid organization within the RBC cytoplasmic membrane

The lipid organization within the RBC_*cm*_ is largely affected by the distribution of head groups, the degree of saturation of the fatty acid tails and the concentration of sterols, such as cholesterol. The molecules are asymmetrically distributed between the two leaflets (Mohandas and Gallagher, 2008). This assymetry is maintained by flippase (Devaux et al., 2008), floppase, and scramblase (Bevers et al., 1999; Zwaal et al., 2005; Daleke, 2008) proteins. PC and SM lipids are predominantly found in the outer leaflet of the membrane while the majority of PE and PI lipids, as well as, all PS and PG lipids are located on the inner leaflet (Mohandas and Gallagher, 2008). Little is known about the distribution of cholesterol within the RBC_*cm*_. It is well established that the molecule can diffuse between both leaflets. Cholesterol flip-flop between the membrane leaflets is an active field of research. Flip-flop rates determined from MD simulations are typically in the microsecond to millisecond time range (Gu et al., 2019; Baral et al., 2020). These rates are typically determined from all-atom simulations that run for tens of *μ*s and by dividing the number of cholesterol molecules that have flipped between the leaflets during that period by the total number of cholesterol molecules in the system. Rates in the order of 10 × 10^4^ s^−1^ are obtained, at the sub-millisecond time scale, with about 40% of molecules undertaking a flip, and most of the flip-flop events take less than ∼50  ns. Millisecond all-atom simulations can be run but are still scarce in the literature, however, it thus seems that longer simulations will not change these rates.

While several experiments confirm these findings (Steck et al., 2002; Bruckner et al., 2009), recent neutron scattering experiments determined flip-flop rates of tens to hundreds of minutes (Garg et al., 2011). Most of these studies were performed on membranes with a simple composition (less than 5 lipid species). The slower cholesterol flipping rates would explain the higher cholesterol concentration in the cytoplasmic leaflet over that in the extracellular leaflet (Gibson Wood et al., 2011), however, are in contradiction to other studies in the literature. Experiments on multicomponent and more complex membranes, such as the RBC_*cm*_, are missing at this point, likely due to experimental challenges. In any case, the flip flop rate was found to be strongly dependent on the membrane’s composition and especially the presence of polyunsaturated lipids substantially increased flip-flop rates. The closest approximation to a RBC_*cm*_ was reported in a recent study by Baral et al. with an all-atom MD model of a RBC_*cm*_ mimic, and flip flop rates between 1.6 × 10^3^–1.9 × 10^4^ 1/s have been determined (Baral et al., 2020). This is interesting, as it quantifies the highly mobile nature of cholesterol within the RBC_*cm*_. At the same time, such a high flip-flop rate suggests a rapid equilibration of the distribution within the membrane. In the absence of any active transport process, one can thus likely assume cholesterol being symmetrically distributed between both leaflets. We note that the apparent contradiction between the flip-flop rates and its biological impact, has yet to be resolved.

The membrane’s asymmetry can be impaired in various conditions. Of particular importance is the exposure of PS on the outer leaflet as it is a strong marker for cell eryptosis (Franck et al., 1985; Nguyen et al., 2011; Vermes et al., 1995; Daleke, 2008). This change in the membrane’s asymmetry is regulated by three mechanisms (Nguyen et al., 2011): 1) Ca^2+^-stimulated scramblase activation (and flippase inhibition) by lysophosphatidic acid, 4-bromo-A23187, and phorbol-12 myristate-13 acetate; 2) protein kinase C activation by lysophosphatidic acid and phorbol-12 myristate-13 acetate; and 3) enhanced lipid flop caused by lysophosphatidic acid. Such molecular changes are often probed in flow cytometry experiments (Vermes et al., 1995) where the Ca^2+^ dependent PL-binding protein Annexin V is used to detect exposed external PS.

While these high-throughput measurements allow for an indirect measurement of the molecular composition and structure of the RBC_*cm*_, analysis of the organization of the individual components requires high resolution biophysical techniques, such as high resolution microscopy (Mikhalyov and Samsonov, 2011; Ciana et al., 2014) or electron microscopy (Liu et al., 1987). Scattering techniques, and especially X-ray diffraction, have become standard methods to investigate membrane structure on molecular length scales. The first X-ray diffraction studies on human erythrocyte membranes date back to 1970 (Knutton et al., 1970; Stamatoff et al., 1975; McCaughan and Krimm, 1980), where RBC ghosts, i.e., empty RBC vesicles, were prepared using the Dodge protocol (Dodge et al., 1963) and pellets of the final preparation were measured. Lamellar periodicities between ≈ 55 and ≈ 70 Å were observed for hemoglobin free membranes. Large amounts of hemoglobin were reported to result in much larger lamellar periodicities of ≈ 110 Å (Knutton et al., 1970). Early electron density profiles predicted a head-to-head thickness of 44 Å (Stamatoff et al., 1975). However, the low purity and low degree of order in the RBC pellets likely prohibited a more detailed structural analysis at this time.

**FIGURE 3:**
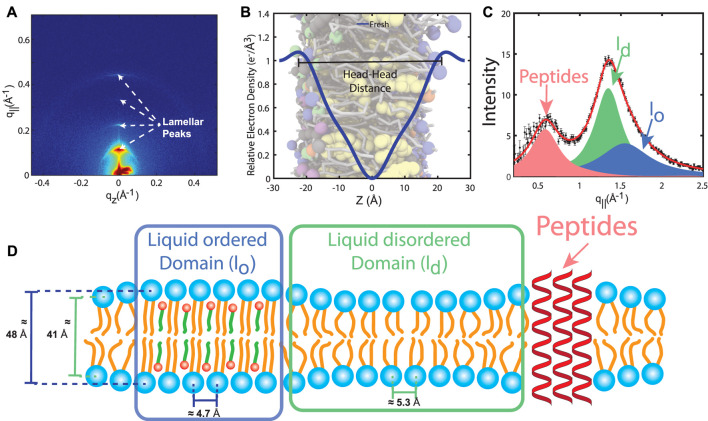
**(A)** Two-dimensional X-ray intensity map recorded on a stack of RBC_*cm*_, measured at 37°C and 88% relative humidity. The lamellar peaks are the result of the lamellar stacking of the membranes. **(B)** Electron density profile reconstructed from the specular peak intensities. The profile is characterized by a global minimum in the bilayer center and two maxima that indicate the location of the electron rich phosphate group of PLs and SMs. **(C)** In-plane X-ray scan reveals three signals that can be assigned to liquid ordered *l*_*o*_, liquid disordered *l*_*d*_ and peptide domains. **(D)** Schematic of the RBC_*cm*_ structure, as determined from the high-resolution X-ray diffraction measurements.

X-ray diffraction experiments are typically conducted on stacks of RBC_*cm*_s applied on solid substrates, such as silicon wafers (Nagle and Tristram-Nagle, 2000; Reviakine and Brisson, 2000; Chen et al., 2001; Rheinstädter et al., 2004; Daillant et al., 2005; Tanaka and Sackmann, 2005; Rheinstädter et al., 2006; Zhou et al., 2007; Pabst et al., 2010; Kučerka et al., 2011; Himbert et al., 2017; Himbert et al., 2020; Himbert et al., 2021). The periodic arrangement of the membranes on the chip maximizes the amount of structural information that can be collected, similar to protein crystallography. A typical high-resolution X-ray diffraction scan of stacked RBC_*cm*_ is shown in Figure  3A. Most scattering signal is specular (*q*_‖_ = 0 Å^−1^) and a series of pronounced lamellar peaks is apparent. These peaks are the results of the periodic stacking of the membranes and the lamellar spacing *d*_*z*_ = 2*π*/*q* can be determined from the peak position. This lamellar spacing comprises the thickness of a single membrane layer plus the thickness of a surrounding water layer that separates the membranes within the stack. Each lamellar peak represents a measurement of the membrane’s form factor, i.e., the Fourier representation of the membrane’s electron density, at a discrete point in the reciprocal space. This allows calculating the RBC_*cm*_’s electron density profile from the specular X-ray scattering through a one-dimensional Fourier analysis, as for instance detailed in (McCaughan and Krimm, 1980; Nagle and Wiener, 1989; Barrett et al., 2012; Himbert et al., 2017; Himbert et al., 2020; Himbert et al., 2021). A typical electron density profile of the RBC_*cm*_ is shown in Figure  3B. These electron density profiles show a central minima in the bilayer center (*z* = 0  Å) and two maxima (|*z*| ≈ 22  Å) which originate from the electron rich phosphate group of PLs and SM. The membrane thickness, *d*_*HH*_, and the thickness of the water layer, *d*_*w*_ = *d*_*z*_ − *d*_*HH*_, can be determined from the distance of the peaks. Values reported in the literature range from *d*_*HH*_ = 39 to 48  Å (Himbert et al., 2017; Himbert et al., 2020; Himbert et al., 2021).

While specular X-ray diffraction signal provides structural information along the bilayer normal, in-plane scattering (Figure  3C) allows measuring distances between neighboring lipids, as well as, the area per lipid tail. Three in-plane signals can be observed in RBC_*cm*_ samples, and can be assigned to two lipid domains which are interpreted as manifestations of liquid ordered *l*_*o*_ and liquid disordered *l*_*d*_ phases, and *α*-helical coiled-coil peptide domains (integral proteins), at ratios of 30.2% *l*_*o*_, 45.0% *l*_*d*_ and 24.8% coiled peptides (Himbert et al., 2017) (Figure  3D). The *l*_*o*_ domains were found to be thicker (*d*_*HH*_ = 48  Å), with more densely packed lipid tails (area per lipid tail, *A*_*T*_ = 19 Å^2^) (Himbert et al., 2017). In contrast, the *l*_*d*_ domains are significantly thinner (41  Å) with a greater lipid tail area (*A*_*T*_ = 25 Å^2^), typical for a fluid structure (Himbert et al., 2017). The average thickness of the peptide domains of 40.6  Å is compatible with the thickness of the membranes and supports the assignment to integral peptides. The patch sizes of both lipid domains are small, on molecular length scales, about 20 and 30  Å (Himbert et al., 2017). The experimentally determined structure of the RBC_*cm*_ is sketched in Figure  3D).

The observation of such heterogeneities is interesting as it agrees well with the raft hypothesis (Simons and Ikonen, 1997; Simons and Ikonen, 2000; Lingwood and Simons, 2009; Simons and Gerl, 2010). Cholesterol is preferably located in areas with saturated lipid tails where it straightens the lipid tails and leads to a reduced area per lipid (Pan et al., 2008). These cholesterol-rich patches are referred to as *rafts*, which are a manifestation of the *l*_*o*_ lipid phase (Simons and Gerl, 2010). The rigid cholesterol molecule has indeed been found to form patches with increased lipid tail order within lipid bilayers (Armstrong et al., 2014; Armstrong et al., 2013; Levental et al., 2020; Nickels et al., 2019; Rheinstädter and Mouritsen, 2013a; Toppozini et al., 2014), surrounded by *l*_*d*_ domains (Simons and Gerl, 2010). The de-mixing of lipid molecules and the formation of lipid rafts is now well established in synthetic lipid membranes that contain saturated, unsaturated PLs and cholesterol (often referred to as raft-forming mixture) (Kučerka et al., 2010; Rheinstädter and Mouritsen, 2013b; Nickels et al., 2019). Lipid rafts are speculated to be relevant for cell signaling events. Properties and even existence of rafts in biological membranes are, however, a topic of intense debate in the literature (Simons and Gerl, 2010; Rheinstädter and Mouritsen, 2013b; Richard et al., 2016). The reason is that we picture rafts as very small, nanometer sized, and highly dynamic structures, which are very difficult to observe (Armstrong et al., 2014; Toppozini et al., 2014). While rafts were initially pictured as more solid patches floating in a less well ordered, fluid membrane environment, in our current understanding, rafts are the result of temporal and spatial inhomogeneities in the membrane (similar to a microemulsion), which form and dissolve spontaneously through the motion of membrane molecules, which is determined by their properties (such as degree of saturation, head group properties), but also topological properties of the membrane, such as curvature or asymmetry (Meinhardt et al., 2013; Sadeghi et al., 2014; Toppozini et al., 2014; Schick, 2019).

Membrane domains have been reported in the RBC_*cm*_. They have been shown to regulate the entry of malarial parasites through mediating the *β*2-adregenic receptor signaling and increasing cAMP levels (Harrison et al., 2003). Techniques such as detergent-resistant-membranes (DRM) (Ciana et al., 2014) or fluorescent labeling (Mikhalyov and Samsonov, 2011; Ciana et al., 2014), but also atomic force microscopy on whole cells in physiological buffer support the presence of rafts in RBCs (Cai et al., 2012). The size of the reported membrane domains ranged between 100 and 300 nm (Cai et al., 2012). This is well below the resolution limit of optical microscopes, however, significantly larger than the speculated size and dynamic range of rafts (Ciana et al., 2014).

Modern *in-silico* methods open new possibilities to investigate mixing and demixing processes within the RBC_*cm*_. Previous MD simulations on large scale models show a similar de-mixing of lipid species in biological cell membranes. Ingolfsson and co-authors demonstrated that these patches form and disappear on nano to microsecond time scales (Ingólfsson et al., 2014). These fluctuations typically average out on longer length and time scales leading to a uniform membrane structure. The observed small dynamic domains are, therefore, not the result of a static phase separation between both membrane species but the result of nanoscopic molecular fluctuations (Ingólfsson et al., 2014; Levental et al., 2016; Ingólfsson et al., 2017; Marrink et al., 2019; Ingólfsson et al., 2020). We note that typical lipid diffusion constants are in the order of 60 × 10^−12^ m^2^/s and the underlying time scale is picoseconds. The lifetime of these patches is therefore about 1,000 times longer than the typical lipid diffusion time scale (Armstrong et al., 2011).

## Mechanical properties of the RBC’s cytoplasmic membrane

**FIGURE 4:**
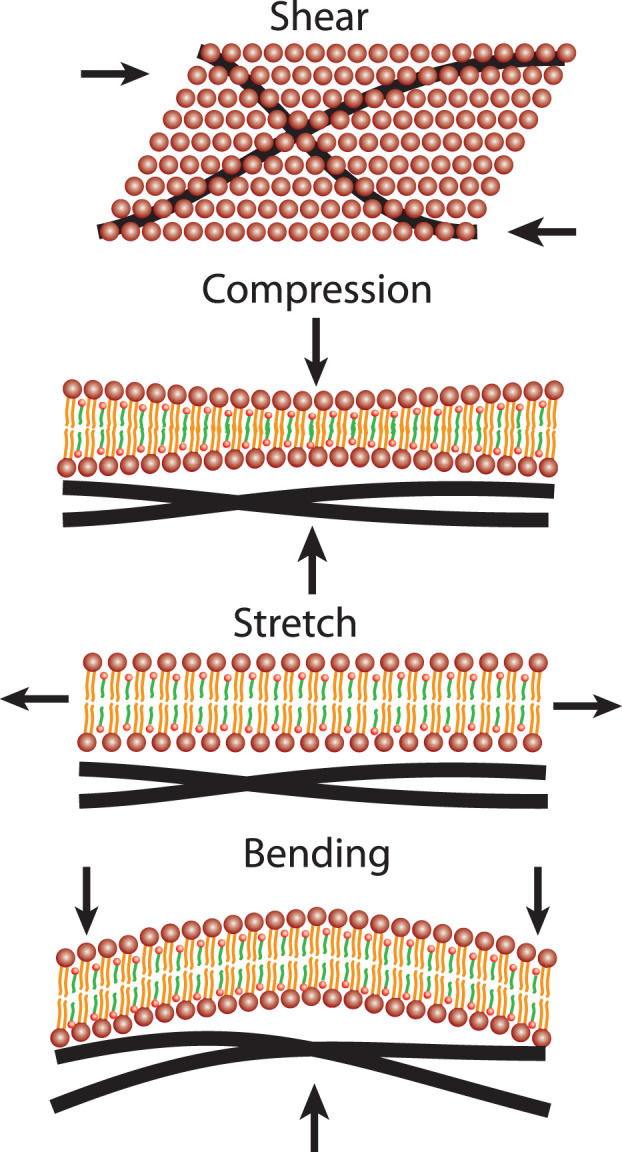
Four deformations of the RBC’s outer shell need to be considered for cells in flow: Shear stress, out-of-plane compressional forces, in-plane stretching forces as well as membrane bending.

RBCs are known for their high deformability. The cells are constantly exposed to mechanical stress as they pass through the vascular system. It is remarkable that the cells can pass through constrictions that are much smaller than their own diameter. Considering that RBCs make up ≈ 45% of the blood’s volume, it is little surprising that the cells mechanical properties strongly correlate with blood’s hydrodynamic properties. When discussing the deformation of cells, there are four distinct forms of mechanical stress: out-of-plane compressional forces, bending forces, shear stress and in-plane compression forces. These four deformation modes are sketched in Figure 4.

The unusual deformability of the RBC’s outer shell is the result of softness combined with a resistance against shear stress and stretching. It is inherently related to the shell’s two layer nature. The shear stability is predominently provided by the cytoskeleton. The network can locally be sheared or stretched by the relative displacement of anchor proteins, while the total surface area of the membrane remains constant. The spectrin network is believed to be slightly stretched in the resting biconcave shape and to exert a compression force on the lipid bilayer (Viallat and Abkarian, 2014). The cytoskeleton elastic response displays strain-stiffening effects (Viallat and Abkarian, 2014). At small deformation, its shear modulus is of the order of 2.5 pN/*μ*m (Viallat and Abkarian, 2014). Spectrin typical bond stretching energies are of the order of k_B_T (Auth et al., 2007) and bond lengths are in the range of 80 nm (Liu et al., 1987). The cytoskeleton’s constant bending modulus is small compared to the typical bending moduli of lipid bilayer membranes (Auth et al., 2007). On the other hand, the RBC_*cm*_ is primarily composed of a lipid bilayer. As such the RBC_*cm*_ is characterized by a bending and in-plane stretch rigidity and vanishing shear rigidity. The RBC_*cm*_ is characterized by a high viscosity (0.1–1 Pa⋅s) (Viallat and Abkarian, 2014).

A simple model of the deformation of lipid bilayers are lipid molecules connected by springs between neighboring molecules, and leaflets that undergo compression and stretching when the membrane is deformed (Phillips et al., 2012). In-plane compression can then be modeled as the compression of springs between the lipid molecules. The corresponding energy cost can be expressed as (Phillips et al., 2012):

$$F_{\mathrm{Compress}}=\frac{K_{A}}{2}\frac{\Delta a^{2}}{a_{0}},$$ (1)

where Δ*a* refers to the change of the membrane area relative to the uncompressed area *a*_0_. *K*_*A*_ is a material property known as the area compression modulus (Phillips et al., 2012). *F*_*Compress*_ has the unit of an energy and *K*_*A*_ has the unit of *force/length*. *K*_*A*_ can be understood as a measure of the force needed, per dimension, to compress a lipid bilayer by a certain length.

The in-plane compression deforms both leaflets evenly. This changes when a bilayer is bent from its flat state. Bending requires an uneven stretching and compression of springs along the out-of-plane coordinate. This can be understood by springs that are compressed in the lower leaflet, while those in the upper leaflet are stretched. The energy cost resulting from bilayer bending is described by (Phillips et al., 2012):

$$F_{\mathrm{Bend}}=\frac{\kappa }{2}\int_{A}da{\left(\nabla^{2}u\left(x,y\right)\right)}^{2},$$ (2)

where *u*(*x*, *y*) describes local spatial deviations of the bilayer center in the out-of-plane direction, *κ* is the membrane’s bending modulus, and *A* is the area covered by the membrane. Eq.  2 is the Helfrich-Canham-Evans functional (Phillips et al., 2012). *κ* is a material property that measures the amount of energy that is needed to bend a membrane. Both *K*_*A*_ and *κ* characterize the membrane’s deformability and several experimental methods have been developed to measure these quantities.

**FIGURE 5:**
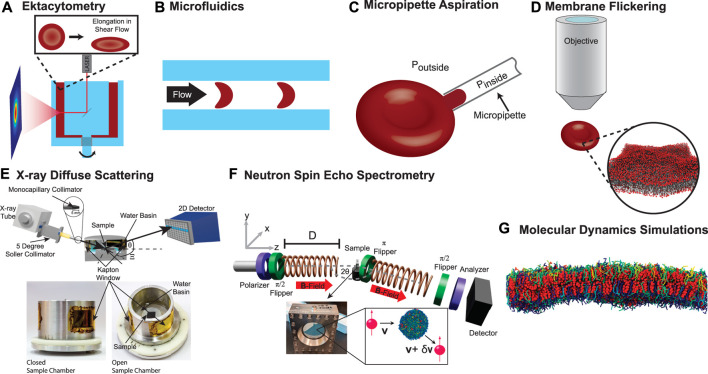
RBC’s deformability can be probed in different techniques: **(A)** Ektacytometry optically probes the elongation of RBC in shear flow. **(B)** The RBC’s deformability can also be determined by visually inspecting single-cell elongation when the cells pass through microcapillaries of a microfluidic device. **(C)** In Micropipette aspiration, a small bulge is formed by means of a pressure gradient between the pipette and the surrounding medium. The deformation is then visually inspected under a microscope and material properties can be determined from a shape analysis. **(D)** Membrane fluctuations can be observed optically and the fluctuation spectrum can be calculated and allows the determination of the membrane’s material characteristics. **(E)** X-ray diffuse scattering probes the membrane’s bending modulus of solid supported, tension-free RBC_*cm*_. Undulating membranes result in off-specular diffuse scattering signal that varies with the membrane’s mechanical properties. **(F)** Neutron spin echo spectrometry probes the membrane’s dynamical properties by measuring the energy transfer of scattered neutrons. **(G)** Molecular Dynamics (MD) simulations allow direct access to the molecules trajectories and fluctuation spectra.

A particularly popular technique is ektacytometry. For this technique, an RBC concentrate is filled into the gap between two concentric transparent cylinders (Figure 5A). A laser beam is guided through the RBC solution and scattered. The diffraction pattern is recorded by a CCD camera outside the outer cylinder and provides a quantitative analysis of the cell’s shape. The cells are then exposed to a shear flow by rotating the inner cylinder and cell elongation is measured from relative changes in the measured diffraction pattern. The elongation allows conclusions on the cell mechanical properties. Importantly, ektacytometry measures an ensemble average and information about individual cells is not recorded. This disadvantage can be overcome by a microfludic approach. Several methods have been employed in the literature such as wedging in tapered constrictions (Gifford et al., 2003) or by measuring the transit time through constrictions by means of pressure change (Abkarian et al., 2006) or electrical impedance (Zheng et al., 2013). An excellent overview over the different methods is presented in a recent review article (Matthews et al., 2022). The method is visualized in Figure 5B. The single-cell elongation in a micro channel with a rectangular cross-section (dimensions between 7 and 15 *μ*m) is visually determined through microscopic techniques. The shape of the RBC depends on the membrane’s deformability and may be quantified through a deformability index (Cluitmans et al., 2014). Recent approaches also enable the in-flow three-dimensional characterization of RBC (Quint et al., 2017) and quantify the observed shapes using machine learning algorithms and demonstrated the importance of the RBC’s deformability for the diagnosis of chorea acanthocytosis/VSP13A disease, McLeod Syndrome or the quality control of stored RBC (Kihm et al., 2018; Recktenwald et al., 2022; Simionato et al., 2021). Another common technique is micropipette aspiration (MA), which can be applied to whole cells and artificially formed liposomes (Figure 5C). A small bulge is formed by sucking a section of the membrane into a micropipette with an opening of a few micrometers. This deformation is then visually inspected under a microscope and both material properties can be determined from a shape analysis of the formed bulge (Evans et al., 1976; Evans, 1983; Evans and Rawicz, 1990; Evans et al., 2008; Dimova, 2014). This approach is taken further by the formation of membrane nanotubes (NT). A nanometer-sized section (diameter ≈ 100 nm) of the membrane is pulled out of a liposome with an optical tweezer and *κ* can be determined from the applied pulling force (Li et al., 2011; Campillo et al., 2013; Dimova, 2014).

The membrane’s elastic properties can also be measured indirectly from a spectral analysis of flickering of cells under a microscope (Brochard and Lennon, 1975; Zilker et al., 1992; Strey et al., 1995), as well as optical interferometric techniques (Popescu et al., 2006; Park et al., 2010b), shown in Figure 5D. Methods that have been employed only recently include X-ray diffuse scattering (XDS), neutron spin echo (NSE) spectrometry and MD simulations. These methods probe the mechanical properties of the RBC’s cytoplasmic membrane on length scales of less than 80 nm, between the pillars of the spectrin network.

In XDS (Kučerka et al., 2006; Tristram-Nagle et al., 2010), elastic properties are determined by diffuse X-ray scattering, which is the result of thermal membrane fluctuations. While (static) structure is typically determined by Bragg scattering, diffuse scattering occurs when molecules move, and scattering occurs at positions away from the resting position. The envelope of diffuse scattering then allows the modeling of dynamical processes. As diffuse scattering is typically orders of magnitude weaker than Bragg scattering, these measurements can only be done at synchrotron X-ray sources or very powerful rotating anode in-house sources. As shown in Figure 5E, solid supported stacks of membranes are used to increase the diffuse signal. In contrast to protein crystallography for instance, membranes must be measured at high temperature (>30°C) and highly hydrated in a humidity chamber (at 99.999% relative humidity). The elastic properties are then determined by fitting the diffuse scattering intensity to a model that calculates the XDS from the membranes’ height-height correlation function. It should be noted that this process is mathematically and computationally intensive and requires high powered GPU supported computers (Himbert, 2021).

These techniques model membrane fluctuations as thermally activated and will deliver incorrect results in the presence of non-equilibrium, activated processes. In this context, ATP has sometimes been thought to affect membrane fluctuations (Betz et al., 2009; Park et al., 2010a), but not always (Evans et al., 2008). Non-thermal noise within experimental error was found at some point from optical tweezer experiments, which could hint to non-thermal sources of membrane motion (Yoon et al., 2011). Turlier et al. (Turlier et al., 2016) more recently reported a violation of the fluctuation–dissipation relation from active and passive microrheology, as a result of the non-equilibrium nature of flickering and the existence of active fluctuations. We note that the experiments discussed below have all been conducted in the explicit absence of ATP such that the origin of the observed fluctuations can be considered as purely thermal.

Relaxations due to membrane undulations on small unilamellar liposomes can be measured directly by inelastic neutron scattering using NSE spectrometry (Rheinstädter et al., 2006; Nagao et al., 2017; Kelley et al., 2020). The corresponding relaxation times are in the order of tens of nanoseconds, with excitation energies in the nano-electron volt range. To measure such a small energy transfers, the velocity of neutrons before and after the interaction with RBC liposomes must be determined with neV precision. To do so, the number of precessions of the neutron spin in a well defined and homogenous magnetic field is “counted” before and behind the sample. A schematic is shown in Figure 5F. With that, the energy transfer between neutron and membrane can be determined as a function of scattering vector, i.e., length scale. The membrane bending rigidity, *κ*, can then be calculated by measuring the membrane relaxation on different length scales. This technique has in the past been limited to artificial lipid bilayers (Pabst et al., 2010; Bouvrais, 2012; Nagle et al., 2015), as they either require a large volume (a typical NSE sample consists of 20 ml with a membrane mass concentration of ≈ 20 mg/ml). The latest generation of NSE instruments now allows to apply this technique to RBC_*cm*_ liposomes, which are available in small concentrations, only, and produce smaller scattering signals due to the increased complexity of real biological membranes, as compared to synthetic bilayers (Himbert et al., 2022). Importantly, the membrane composition and especially the presence of cholesterol has to be considered (Nagle, 2021; Nagle et al., 2021) in the analysis.

With the ever-increasing computing power and refined algorithms, MD simulations have become indispensable tools in biomedical research. While experiments are typically done using a large number of molecules (at millimolar concentrations equivalent to $\approx 10^{20}$ molecules), and measured values are thus the result of large ensemble averages, MD simulations can complement experiments by providing a high-resolution view on certain processes, which is essential to decipher the underlying molecular-mode-of-action. Because modern computers can run realistic simulations of high complexity and large system size (in particular using coarse-grained simulations), MD simulations can quantitatively be compared to experimental results. Simulations of biological membranes have been presented relatively recently, and studies are still scarce. Simulations of RBC_*cm*_ can now deliver unprecedented details of their structure and lipid organization, but also details on the interaction with membrane-active molecules. A snapshot of such a coarse-grained Martini simulation is shown in Figure 5G. Membrane undulations can be studied from the time trajectories of the simulations, typically over tens of *μ*s, and the bending rigidity can be determined from the fluctuation spectrum.

Table 1 lists values for the bending rigidity, *κ*, and the area compression modulus, *K*_*A*_, for RBCs and RBC_*cm*_. Values for *κ* are reported over a wide range, from 210 k_B_T down to 2 k_B_T and the disparate experimental results have been appropriately described as puzzling (Strey et al., 1995; Auth et al., 2007). It was suggested that these apparent controversial results can be explained by the complex interplay between the membrane bilayer and the spectrin network (Strey et al., 1995). Correlation of the magnitude of *κ* with the length scale of the experiments has been suggested (Lipowsky and Girardet, 1990; Auth et al., 2007), and one would associate a crossover length scale with the 80 nm mesh of the spectrin network. At length scales substantially greater than that, the composite RBC shell is homogeneous and would be characterized by a bending modulus for both the cytoplasmic membrane and the spectrin network.

**TABLE 1:** Summary of values reported for the bending rigidity, *κ*, and the area compression modulus, *K*_*A*_, of discocytic RBCs and RBC_*cm*_. Also included is an estimate of the length scale on which the different techniques work.

Technique	*κ* (k_B_T)	Lengthscale	*K*_*A*_ (mN/m)		Reference
		(*μ*m)			
Flickering Analysis	3–9	> 0.6		RBC	Brochard and Lennon, (1975)
Micropipette Aspiration Buckling	43	> 7		RBC	Evans, (1983)
Reflection Interference Microscopy	5±1.5	> 0.25		RBC	Zilker et al. (1992)
Reflection Interference Microscopy	97±37	> 2		RBC	Strey et al. (1995)
Diffraction Phase Microscopy	16±0.3	> 0.1		RBC	Popescu et al. (2006)
Reanalysis of (Popescu et al., 2006)	14, 25	> 0.1		RBC	Auth et al. (2007)
Flickering Analysis	210	> 0.7		RBC	Evans et al. (2008)
Optical Tweezer	68±0.68	> 7		RBC	Betz et al. (2009)
Flickering Analysis	67±13	> 1.5		RBC	Yoon et al. (2009)
Diffraction Phase Microscopy	7±3	> 0.1		RBC	Park et al. (2010b)
Diffraction Phase Microscopy	5±2	> 0.1		RBC	Park et al. (2011)
Diffuse X-ray Scattering	2–6	< 0.08		RBC_*cm*_	Himbert et al. (2022)
Neutron Spin-Echo	4–7	< 0.08		RBC_*cm*_	Himbert et al. (2022)
Molecular Dynamics	4	< 0.08		RBC_*cm*_	Himbert et al. (2022)
Micropipette Aspiration			288±50	RBC	Evans et al. (1976)
Micropipette Aspiration			450	RBC	Evans and Waugh, (1977)
Diffraction Phase Microscopy			15.5 × 10^−3^ ± 2.5 × 10^−3^	RBC	Park et al. (2010b)

Measurements on the length scale of the whole cell, such as buckling in an aspiration pipette experiment (43 k_B_T) (Evans, 1983) and deformations induced by optical tweezers (*κ* = 67 k_B_T (Betz et al., 2009)) would provide these values. For length scales smaller than the crossover length scale, most of the bending would be situated in the cytoplasmic membrane between the ribs of the spectrin network, and *κ* values from measurements on those length scales would approach those of just the cytoplasmic membrane, which is likely to be homogeneous down to a length scale of 10 nm. However, measurements on molecular length scales do not support the preceding scenario. The smaller values in Table 1 by Brochard et al. [*κ* = 3–9 k_B_T (Brochard and Lennon, 1975)], and Park et al. [*κ* = 7 k_B_T (Park et al., 2010b)], Zilker et al. [*κ* = 5 k_B_T (Zilker et al., 1992)] are from measurements with length scales of the order of the wavelength 400 nm of the optical methods employed. That is not smaller than the spectrin network length scale of 80 nm, so crossover to the value of *κ* for the cytoplasmic membrane would only be expected to have just begun. Instead, those *κ* values are much smaller than the values obtained from the largest length scale measurements. At even smaller length scales one would further expect complete crossover to a still smaller value of *κ*. That value would be just that of the cytoplasmic membrane. Contrarily, values for the cytoplasmic membrane from XDS, NSE and MD are roughly equal to the small values obtained at the optical length scales (Brochard and Lennon, 1975; Zilker et al., 1992; Park et al., 2010b; Park et al., 2011). There is thus strong evidence that there is no length scale dependence in *κ*. This implies that there is no contribution of the spectrin network to the RBC bending modulus, in agreement with (Gov et al., 2003). The bending modulus of the RBC_*cm*_ is relatively small, in the range of 4 k_B_T to 6 k_B_T. Even though this is a rather large uncertainty range, it is still significant and points to an extreme softness of RBCs on small length scales. The only conclusions that can be drawn by reviewing the existing literature at this point seem to be 1: The bending rigidity of red blood cells is reported over a large range, from 3 k_B_T to about 100 k_B_T, which does not depend on the technique 2: The spread in values cannot be explained by a length scale dependence of *κ*, as techniques which measure on large length scales report small and large values and vice versa 3: Techniques that isolate the RBC cytoplasmic membrane and only measure *κ* of the RBC_*cm*_ report very small values, between 4–6 k_B_T 4: Because of these measurements, the contribution of the spectrin network is likely negligible as there are measurements of entire RBCs which also report such small values.

The nanoscopic regime is most relevant for cellular processes which take place between the ribs of the spectrin network. Especially the passive transport of small molecules is intrinsically related to the membrane’s properties on small length scales (Ghysels et al., 2019; Angles et al., 2021). Of course, RBCs are required to efficiently exchange oxygen and carbon dioxide across the membrane. One may speculate that such permeability is enhanced in a softer membrane, and a standard measure of softness is having a smaller bending modulus. As such, a smaller bending modulus of the RBC_*cm*_ would generally indicate physiological advantage. While a correlation between the area compressibility modulus and diffusion is obvious as a larger area creates more free volume for particles to move, a correlation between bending rigidity and passive water diffusion has been found (Mathai et al., 2007). However, no experiments regarding membrane stiffness and gas permeability have been reported at this point.

**FIGURE 6:**
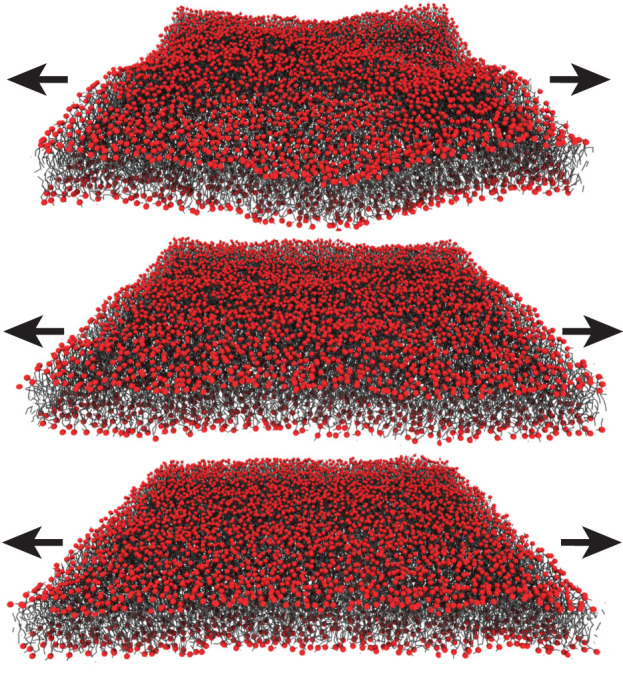
The area compressibility modulus of lipid bilayer is typically a stiff modulus between 200 and 300 mN/m. However, much lower values have been also reported for the RBC_*cm*_ (Park et al., 2010b). This controversy may be explained when considering conformational states of the membrane. A tension free membrane with a low bending rigidity disclose flickering. Wrinkles of the bilayer must be flattened first when stretching the membrane, characterized by a low stretch resistance. In a subsequent regime, the lipid bilayer itself will be stretched which results in a much larger area compressibility modulus *K*_*A*_.

If one accepts a small value of *κ* for the RBC_*cm*_, the question remains if it could be beneficial for biological membranes to be so soft. A possible advantage of a small bending modulus of the cytoplasmic membrane might be that it reduces the energy cost for the process of squeezing the RBC through small capillaries. This hypothesis is based on the possibility that such mechanical processes might require local area changes in the cytoplasmic membrane. Such changes could be slaved to changes in the local area of the spectrin network if the latter changes were required. Even if the spectrin network is rigid with respect to local area changes, changes in its local curvature would necessarily change the local area of the attached cytoplasmic membrane. It is usual to think of the free energy for area change in terms of the area compressibility modulus *K*_*A*_, which is a fairly stiff modulus, typically 250 mN/m. This modulus is associated with area changes per molecule in a flat membrane and the work done to change the molecular packing. However, in the flaccid, low surface tension regime, the membrane has thermally induced undulations that make the cell’s projected area smaller than the local area (Rawicz et al., 2000). Small increases in the tension pulls out these undulations, resulting in an increase in projected area that corresponds to a much smaller apparent *K*_*A*_ than the one usually reported. This process is sketched in Figure 6. Indeed, such an apparent *K*_*A*_ of only 15 *μ*N/m has been reported for the RBC (Park et al., 2010b). This means that there is a regime of area strain that costs very little energy. How far the area can change in this low cost regime varies nearly inversely with the bending modulus *κ*. This regime has been measured to extend up to an area increase of about 2% for a lipid bilayer with *κ* = 10 k_B_T (Rawicz et al., 2000). The smaller *κ* of the RBC_*cm*_ thus increases the low-cost regime and would therefore provide a greater range of mechanical flexibility that may be advantageous for blood flow.

If one accepts the above conclusion that *κ* of the RBC_*cm*_ is small, even 10 times smaller than typical bending rigidities of much simpler synthetic membranes, an important point is the role of cholesterol for the mechanical properties of the RBC_*cm*_. It is interesting that *κ* of the RBC_*cm*_ is so small when it has 50% cholesterol, which is of often found to stiffen bilayers composed of pure lipids while our current belief is that a high concentration of cholesterol leads to stiff membranes. It has been shown that the stiffening effect of cholesterol decreases with increasing unsaturation (Pan et al., 2009) and vanishes already in DOPC and diC22:1PC that have just one unsaturated double bond in each chain. Extrapolation would then suggest that cholesterol might even decrease the bending modulus of membranes with a significant concentration of lipids with multiple double bonds. Cholesterol’s rigid molecular structure contrasts the flexible nature of fatty acyl tails and increases the membrane’s bending modulus in fully saturated model membranes (Pan et al., 2008; Pan et al., 2009). However, little is known about the effect of cholesterol on the bending rigidity of multi-component membranes. Only ≈ 1/3 of the lipids within the RBC_*cm*_ were found to be fully saturated and experiments on synthetic membranes found a negligible effect of cholesterol on the membrane’s bending rigidity when there are unsaturated molecules present.

However, a particularly interesting observation in this context is the increase in both the bending rigidity in MD simulation for which the polyunsaturated lipids or cholesterol have been removed from a RBC_*cm*_ mimic (Himbert et al., 2022), respectively. The absence of either of poly-unsaturated lipids or cholesterol made *κ* increase from 4 k_B_T to about 13 k_B_T. This strongly suggests that the softness of the RBC_*cm*_ can at least partially be explained by the interplay between lipids with higher degrees of tail unsaturation and the stiff cholesterol molecule. While this hypothesis sheds light on some of the confusing results that have been reported in simpler model membranes, it needs to be confirmed in other biological membranes. Cholesterol is an essential component of eucaryotic membranes and makes up to ≈ 50% of the membrane’s content. If it is also true that there is an advantage for biological membranes to be soft at the same time to support membrane diffusion and support cell mechanics, an interaction between unsaturated and cholesterol could provide a missing piece in our current understanding of cell membrane properties.

## Conclusion

Modern biophysical approaches can contribute to our understanding of the red blood cell’s cytoplasmic membrane. They picture the RBC_*cm*_ as patchy, with nanometer sized liquid ordered and disordered lipid and peptide domains. The membrane is at the same time surprisingly soft, with bending rigidities *κ* of 2–6 k_B_T, only. This extreme softness is the result of an interaction between poly unsaturated lipids and cholesterol, and in strong contrast to the current belief that a high concentration of cholesterol in general results in stiff membranes. As permeability is typically enhanced in a softer membrane, a smaller bending modulus of the RBC_*cm*_ would generally indicate a physiological advantage. A small bending rigidity can also reduce the energy for the process of squeezing the RBC through small capillaries by providing a low-cost mechanism, where small increases in the tension pull out membrane undulations, resulting in an increase in projected membrane area without actually stretching the RBC_*cm*_ (which is more costly in terms of energy). By reviewing the current literature, we present convincing evidence that there is no length scale dependence in *κ* of whole RBCs. This also implies that there is no contribution of the spectrin network to the RBC bending modulus.
